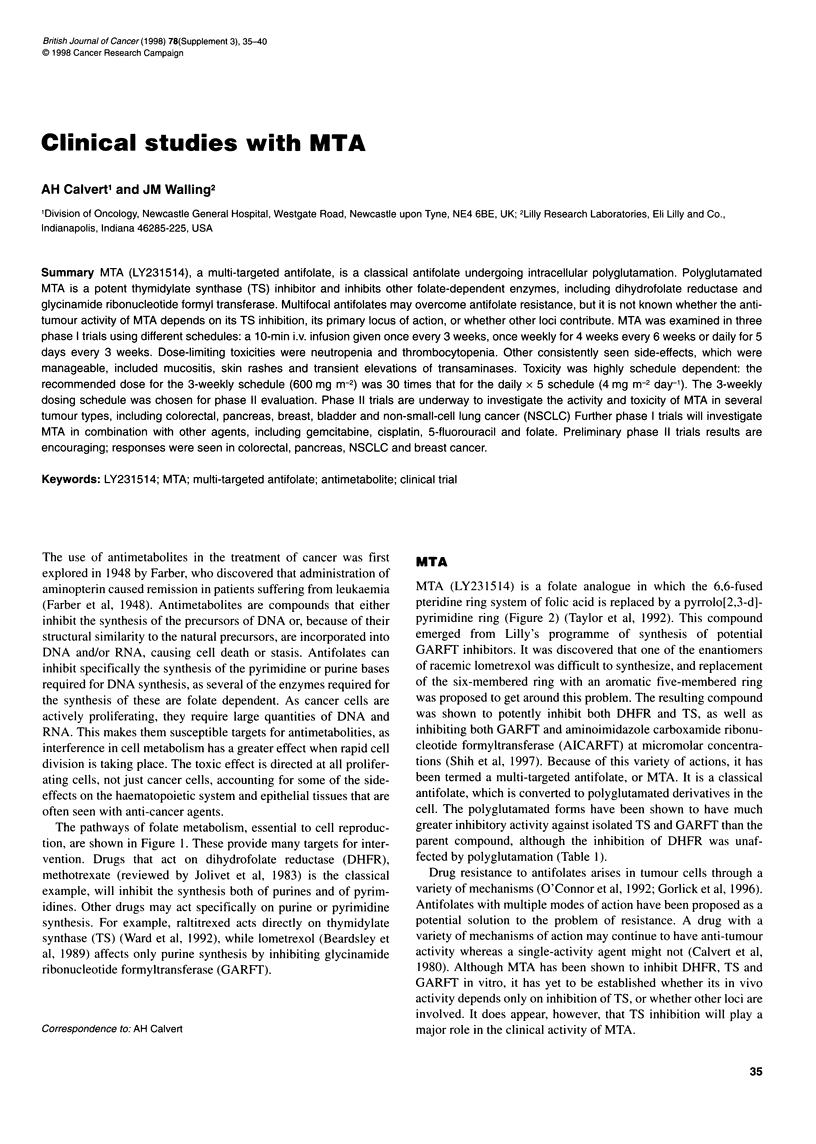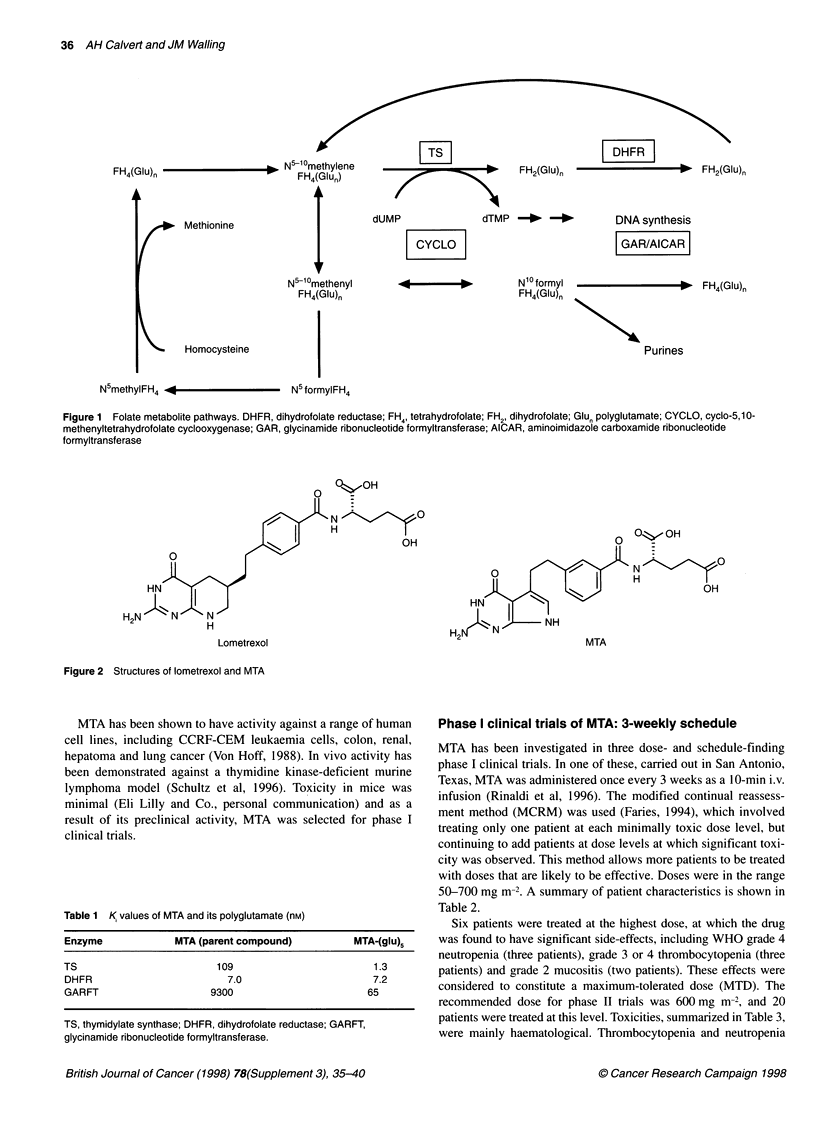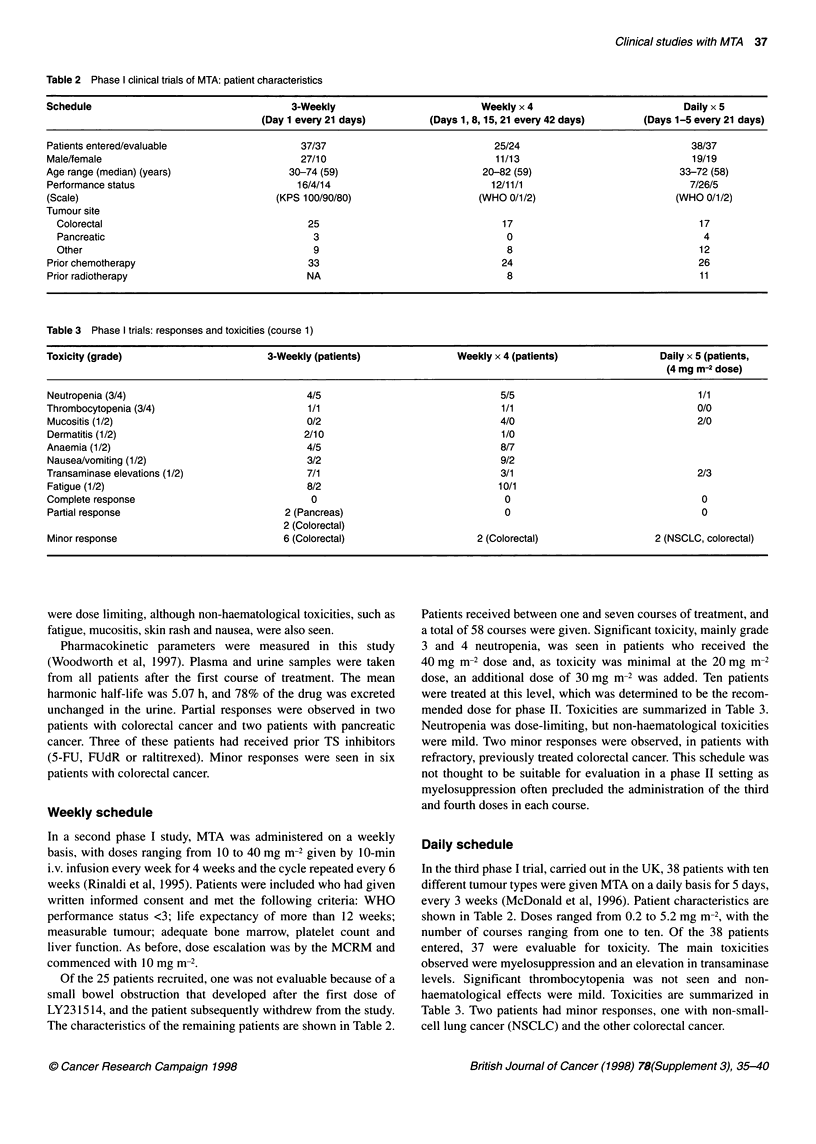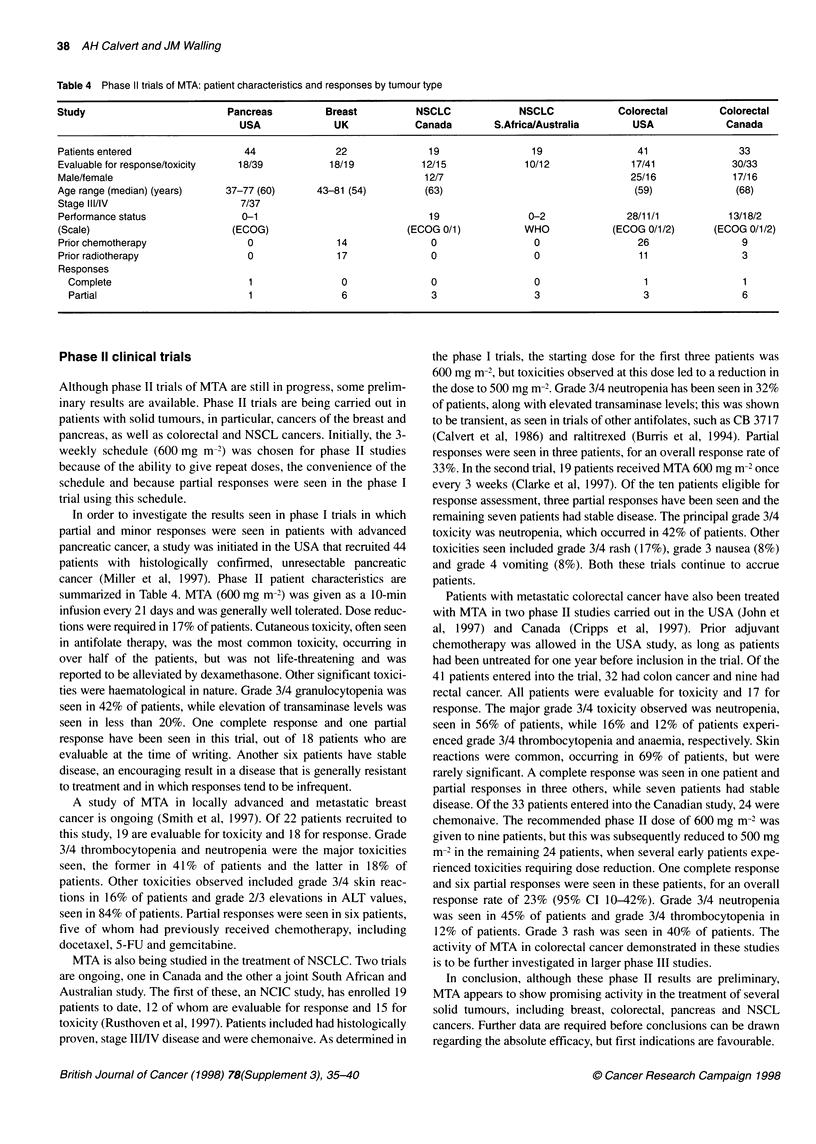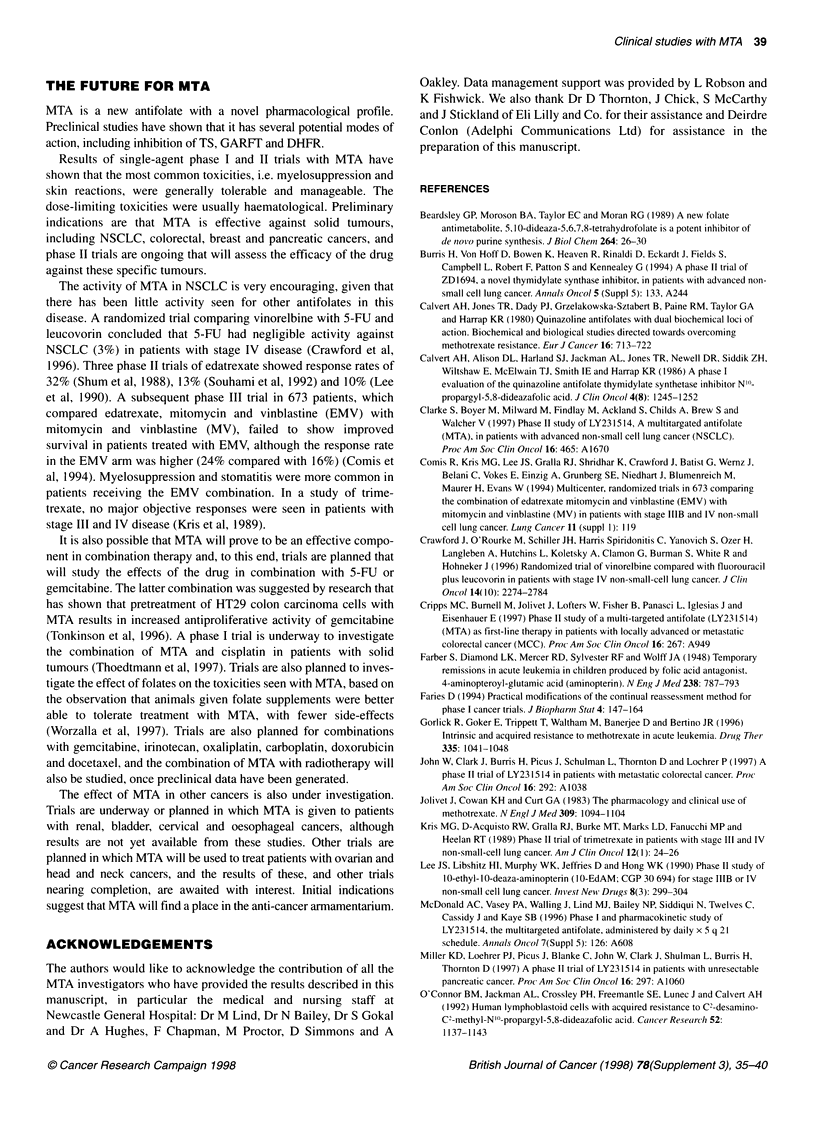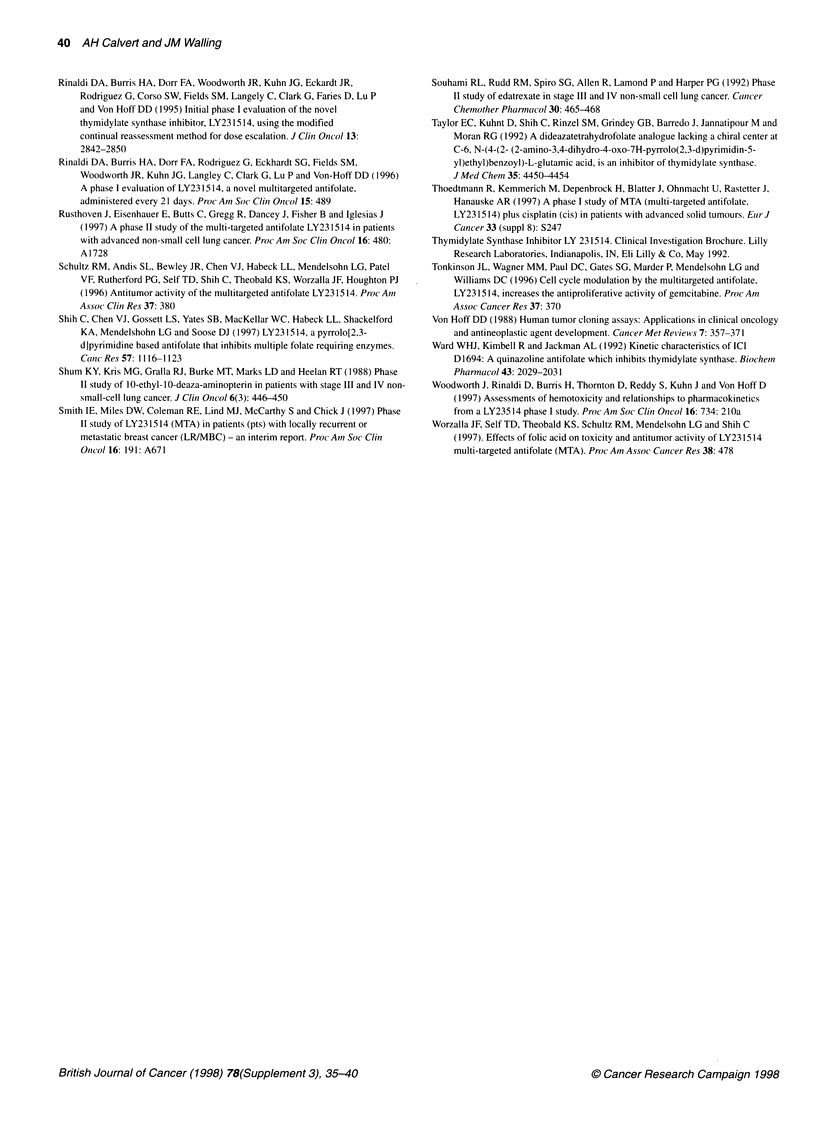# Clinical studies with MTA.

**DOI:** 10.1038/bjc.1998.752

**Published:** 1998

**Authors:** A. H. Calvert, J. M. Walling

**Affiliations:** Division of Oncology, Newcastle General Hospital, Newcastle upon Tyne, UK.

## Abstract

MTA (LY231514), a multi-targeted antifolate, is a classical antifolate undergoing intracellular polyglutamation. Polyglutamated MTA is a potent thymidylate synthase (TS) inhibitor and inhibits other folate-dependent enzymes, including dihydrofolate reductase and glycinamide ribonucleotide formyl transferase. Multifocal antifolates may overcome antifolate resistance, but it is not known whether the anti-tumour activity of MTA depends on its TS inhibition, its primary locus of action, or whether other loci contribute. MTA was examined in three phase I trials using different schedules: a 10-min i.v. infusion given once every 3 weeks, once weekly for 4 weeks every 6 weeks or daily for 5 days every 3 weeks. Dose-limiting toxicities were neutropenia and thrombocytopenia. Other consistently seen side-effects, which were manageable, included mucositis, skin rashes and transient elevations of transaminases. Toxicity was highly schedule dependent: the recommended dose for the 3-weekly schedule (600 mg m(-2)) was 30 times that for the daily x 5 schedule (4 mg m(-2)day(-1)). The 3-weekly dosing schedule was chosen for phase II evaluation. Phase II trials are underway to investigate the activity and toxicity of MTA in several tumour types, including colorectal, pancreas, breast, bladder and non-small-cell lung cancer (NSCLC) Further phase I trials will investigate MTA in combination with other agents, including gemcitabine, cisplatin, 5-fluorouracil and folate. Preliminary phase II trials results are encouraging; responses were seen in colorectal, pancreas, NSCLC and breast cancer.


British Journal of Cancer (1998) 78(Supplement 3), 35–40
© 1998 Cancer Research Campaign

### Clinical studies with MTA

**AH Calvert[1] and JM Walling[2]**

[1]Division of Oncology, Newcastle General Hospital, Westgate Road, Newcastle upon Tyne, NE4 6BE, UK; [2]Lilly Research Laboratories, Eli Lilly and Co., Indianapolis, Indiana 46285-225, USA

**Summary** MTA (LY231514), a multi-targeted antifolate, is a classical antifolate undergoing intracellular polyglutamation. Polyglutamated MTA is a potent thymidylate synthase (TS) inhibitor and inhibits other folate-dependent enzymes, including dihydrofolate reductase and glycinamide ribonucleotide formyl transferase. Multifocal antifolates may overcome antifolate resistance, but it is not known whether the anti-tumour activity of MTA depends on its TS inhibition, its primary locus of action, or whether other loci contribute. MTA was examined in three phase I trials using different schedules: a 10-min i.v. infusion given once every 3 weeks, once weekly for 4 weeks every 6 weeks or daily for 5 days every 3 weeks. Dose-limiting toxicities were neutropenia and thrombocytopenia. Other consistently seen side-effects, which were manageable, included mucositis, skin rashes and transient elevations of transaminases. Toxicity was highly schedule dependent: the recommended dose for the 3-weekly schedule (600 mg $m^{-2}$) was 30 times that for the daily × 5 schedule (4 mg $m^{-2}$ $day^{-1}$). The 3-weekly dosing schedule was chosen for phase II evaluation. Phase II trials are underway to investigate the activity and toxicity of MTA in several tumour types, including colorectal, pancreas, breast, bladder and non-small-cell lung cancer (NSCLC) Further phase I trials will investigate MTA in combination with other agents, including gemcitabine, cisplatin, 5-fluorouracil and folate. Preliminary phase II trials results are encouraging; responses were seen in colorectal, pancreas, NSCLC and breast cancer.

**Keywords:** LY231514; MTA; multi-targeted antifolate; antimetabolite; clinical trial

The use of antimetabolites in the treatment of cancer was first explored in 1948 by Farber, who discovered that administration of aminopterin caused remission in patients suffering from leukaemia (Farber et al, 1948). Antimetabolites are compounds that either inhibit the synthesis of the precursors of DNA or, because of their structural similarity to the natural precursors, are incorporated into DNA and/or RNA, causing cell death or stasis. Antifolates can inhibit specifically the synthesis of the pyrimidine or purine bases required for DNA synthesis, as several of the enzymes required for the synthesis of these are folate dependent. As cancer cells are actively proliferating, they require large quantities of DNA and RNA. This makes them susceptible targets for antimetabolities, as interference in cell metabolism has a greater effect when rapid cell division is taking place. The toxic effect is directed at all proliferating cells, not just cancer cells, accounting for some of the side-effects on the haematopoietic system and epithelial tissues that are often seen with anti-cancer agents.

The pathways of folate metabolism, essential to cell reproduction, are shown in Figure 1. These provide many targets for intervention. Drugs that act on dihydrofolate reductase (DHFR), methotrexate (reviewed by Jolivet et al, 1983) is the classical example, will inhibit the synthesis both of purines and of pyrimidines. Other drugs may act specifically on purine or pyrimidine synthesis. For example, raltitrexed acts directly on thymidylate synthase (TS) (Ward et al, 1992), while lometrexol (Beardsley et al, 1989) affects only purine synthesis by inhibiting glycinamide ribonucleotide formyltransferase (GARFT).

*Correspondence to:* AH Calvert

### MTA

MTA (LY231514) is a folate analogue in which the 6,6-fused pteridine ring system of folic acid is replaced by a pyrrolo[2,3-d]-pyrimidine ring (Figure 2) (Taylor et al, 1992). This compound emerged from Lilly's programme of synthesis of potential GARFT inhibitors. It was discovered that one of the enantiomers of racemic lometrexol was difficult to synthesize, and replacement of the six-membered ring with an aromatic five-membered ring was proposed to get around this problem. The resulting compound was shown to potently inhibit both DHFR and TS, as well as inhibiting both GARFT and aminoimidazole carboxamide ribonucleotide formyltransferase (AICARFT) at micromolar concentrations (Shih et al, 1997). Because of this variety of actions, it has been termed a multi-targeted antifolate, or MTA. It is a classical antifolate, which is converted to polyglutamated derivatives in the cell. The polyglutamated forms have been shown to have much greater inhibitory activity against isolated TS and GARFT than the parent compound, although the inhibition of DHFR was unaffected by polyglutamation (Table 1).

Drug resistance to antifolates arises in tumour cells through a variety of mechanisms (O'Connor et al, 1992; Gorlick et al, 1996). Antifolates with multiple modes of action have been proposed as a potential solution to the problem of resistance. A drug with a variety of mechanisms of action may continue to have anti-tumour activity whereas a single-activity agent might not (Calvert et al, 1980). Although MTA has been shown to inhibit DHFR, TS and GARFT in vitro, it has yet to be established whether its in vivo activity depends only on inhibition of TS, or whether other loci are involved. It does appear, however, that TS inhibition will play a major role in the clinical activity of MTA.

**Figure 1** Folate metabolite pathways. DHFR, dihydrofolate reductase; $FH_4$, tetrahydrofolate; $FH_2$, dihydrofolate; $Glu_n$ polyglutamate; CYCLO, cyclo-5,10-methenyltetrahydrofolate cyclooxygenase; GAR, glycinamide ribonucleotide formyltransferase; AICAR, aminoimidazole carboxamide ribonucleotide formyltransferase

**Figure 2** Structures of lometrexol and MTA

MTA has been shown to have activity against a range of human cell lines, including CCRF-CEM leukaemia cells, colon, renal, hepatoma and lung cancer (Von Hoff, 1988). In vivo activity has been demonstrated against a thymidine kinase-deficient murine lymphoma model (Schultz et al, 1996). Toxicity in mice was minimal (Eli Lilly and Co., personal communication) and as a result of its preclinical activity, MTA was selected for phase I clinical trials.

**Table 1** $K_i$ values of MTA and its polyglutamate (nM)

| Enzyme | MTA (parent compound) | MTA-(glu)$_5$ |
|---|---|---|
| TS | 109 | 1.3 |
| DHFR | 7.0 | 7.2 |
| GARFT | 9300 | 65 |

TS, thymidylate synthase; DHFR, dihydrofolate reductase; GARFT, glycinamide ribonucleotide formyltransferase.

### Phase I clinical trials of MTA: 3-weekly schedule

MTA has been investigated in three dose- and schedule-finding phase I clinical trials. In one of these, carried out in San Antonio, Texas, MTA was administered once every 3 weeks as a 10-min i.v. infusion (Rinaldi et al, 1996). The modified continual reassessment method (MCRM) was used (Faries, 1994), which involved treating only one patient at each minimally toxic dose level, but continuing to add patients at dose levels at which significant toxicity was observed. This method allows more patients to be treated with doses that are likely to be effective. Doses were in the range 50–700 mg m$^{-2}$. A summary of patient characteristics is shown in Table 2.

Six patients were treated at the highest dose, at which the drug was found to have significant side-effects, including WHO grade 4 neutropenia (three patients), grade 3 or 4 thrombocytopenia (three patients) and grade 2 mucositis (two patients). These effects were considered to constitute a maximum-tolerated dose (MTD). The recommended dose for phase II trials was 600 mg m$^{-2}$, and 20 patients were treated at this level. Toxicities, summarized in Table 3, were mainly haematological. Thrombocytopenia and neutropenia

© Cancer Research Campaign 1998

**Table 2** Phase I clinical trials of MTA: patient characteristics

| Schedule | 3-Weekly (Day 1 every 21 days) | Weekly × 4 (Days 1, 8, 15, 21 every 42 days) | Daily × 5 (Days 1–5 every 21 days) |
|---|---|---|---|
| Patients entered/evaluable | 37/37 | 25/24 | 38/37 |
| Male/female | 27/10 | 11/13 | 19/19 |
| Age range (median) (years) | 30–74 (59) | 20–82 (59) | 33–72 (58) |
| Performance status | 16/4/14 | 12/11/1 | 7/26/5 |
| (Scale) | (KPS 100/90/80) | (WHO 0/1/2) | (WHO 0/1/2) |
| Tumour site | | | |
| Colorectal | 25 | 17 | 17 |
| Pancreatic | 3 | 0 | 4 |
| Other | 9 | 8 | 12 |
| Prior chemotherapy | 33 | 24 | 26 |
| Prior radiotherapy | NA | 8 | 11 |

**Table 3** Phase I trials: responses and toxicities (course 1)

| Toxicity (grade) | 3-Weekly (patients) | Weekly × 4 (patients) | Daily × 5 (patients, (4 mg m$^{-2}$ dose) |
|---|---|---|---|
| Neutropenia (3/4) | 4/5 | 5/5 | 1/1 |
| Thrombocytopenia (3/4) | 1/1 | 1/1 | 0/0 |
| Mucositis (1/2) | 0/2 | 4/0 | 2/0 |
| Dermatitis (1/2) | 2/10 | 1/0 | |
| Anaemia (1/2) | 4/5 | 8/7 | |
| Nausea/vomiting (1/2) | 3/2 | 9/2 | |
| Transaminase elevations (1/2) | 7/1 | 3/1 | 2/3 |
| Fatigue (1/2) | 8/2 | 10/1 | |
| Complete response | 0 | 0 | 0 |
| Partial response | 2 (Pancreas)<br>2 (Colorectal) | 0 | 0 |
| Minor response | 6 (Colorectal) | 2 (Colorectal) | 2 (NSCLC, colorectal) |

were dose limiting, although non-haematological toxicities, such as fatigue, mucositis, skin rash and nausea, were also seen.

Pharmacokinetic parameters were measured in this study (Woodworth et al, 1997). Plasma and urine samples were taken from all patients after the first course of treatment. The mean harmonic half-life was 5.07 h, and 78% of the drug was excreted unchanged in the urine. Partial responses were observed in two patients with colorectal cancer and two patients with pancreatic cancer. Three of these patients had received prior TS inhibitors (5-FU, FUdR or raltitrexed). Minor responses were seen in six patients with colorectal cancer.

### Weekly schedule

In a second phase I study, MTA was administered on a weekly basis, with doses ranging from 10 to 40 mg m$^{-2}$ given by 10-min i.v. infusion every week for 4 weeks and the cycle repeated every 6 weeks (Rinaldi et al, 1995). Patients were included who had given written informed consent and met the following criteria: WHO performance status <3; life expectancy of more than 12 weeks; measurable tumour; adequate bone marrow, platelet count and liver function. As before, dose escalation was by the MCRM and commenced with 10 mg m$^{-2}$.

Of the 25 patients recruited, one was not evaluable because of a small bowel obstruction that developed after the first dose of LY231514, and the patient subsequently withdrew from the study. The characteristics of the remaining patients are shown in Table 2. Patients received between one and seven courses of treatment, and a total of 58 courses were given. Significant toxicity, mainly grade 3 and 4 neutropenia, was seen in patients who received the 40 mg m$^{-2}$ dose and, as toxicity was minimal at the 20 mg m$^{-2}$ dose, an additional dose of 30 mg m$^{-2}$ was added. Ten patients were treated at this level, which was determined to be the recommended dose for phase II. Toxicities are summarized in Table 3. Neutropenia was dose-limiting, but non-haematological toxicities were mild. Two minor responses were observed, in patients with refractory, previously treated colorectal cancer. This schedule was not thought to be suitable for evaluation in a phase II setting as myelosuppression often precluded the administration of the third and fourth doses in each course.

### Daily schedule

In the third phase I trial, carried out in the UK, 38 patients with ten different tumour types were given MTA on a daily basis for 5 days, every 3 weeks (McDonald et al, 1996). Patient characteristics are shown in Table 2. Doses ranged from 0.2 to 5.2 mg m$^{-2}$, with the number of courses ranging from one to ten. Of the 38 patients entered, 37 were evaluable for toxicity. The main toxicities observed were myelosuppression and an elevation in transaminase levels. Significant thrombocytopenia was not seen and non-haematological effects were mild. Toxicities are summarized in Table 3. Two patients had minor responses, one with non-small-cell lung cancer (NSCLC) and the other colorectal cancer.

© Cancer Research Campaign 1998

**Table 4** Phase II trials of MTA: patient characteristics and responses by tumour type

| Study | Pancreas USA | Breast UK | NSCLC Canada | NSCLC S.Africa/Australia | Colorectal USA | Colorectal Canada |
|---|---|---|---|---|---|---|
| Patients entered | 44 | 22 | 19 | 19 | 41 | 33 |
| Evaluable for response/toxicity | 18/39 | 18/19 | 12/15 | 10/12 | 17/41 | 30/33 |
| Male/female | | | 12/7 | | 25/16 | 17/16 |
| Age range (median) (years) | 37–77 (60) | 43–81 (54) | (63) | | (59) | (68) |
| Stage III/IV | 7/37 | | | | | |
| Performance status | 0–1 | | 19 | 0–2 | 28/11/1 | 13/18/2 |
| (Scale) | (ECOG) | | (ECOG 0/1) | WHO | (ECOG 0/1/2) | (ECOG 0/1/2) |
| Prior chemotherapy | 0 | 14 | 0 | 0 | 26 | 9 |
| Prior radiotherapy | 0 | 17 | 0 | 0 | 11 | 3 |
| Responses | | | | | | |
| Complete | 1 | 0 | 0 | 0 | 1 | 1 |
| Partial | 1 | 6 | 3 | 3 | 3 | 6 |

### Phase II clinical trials

Although phase II trials of MTA are still in progress, some preliminary results are available. Phase II trials are being carried out in patients with solid tumours, in particular, cancers of the breast and pancreas, as well as colorectal and NSCL cancers. Initially, the 3-weekly schedule (600 mg m$^{-2}$) was chosen for phase II studies because of the ability to give repeat doses, the convenience of the schedule and because partial responses were seen in the phase I trial using this schedule.

In order to investigate the results seen in phase I trials in which partial and minor responses were seen in patients with advanced pancreatic cancer, a study was initiated in the USA that recruited 44 patients with histologically confirmed, unresectable pancreatic cancer (Miller et al, 1997). Phase II patient characteristics are summarized in Table 4. MTA (600 mg m$^{-2}$) was given as a 10-min infusion every 21 days and was generally well tolerated. Dose reductions were required in 17% of patients. Cutaneous toxicity, often seen in antifolate therapy, was the most common toxicity, occurring in over half of the patients, but was not life-threatening and was reported to be alleviated by dexamethasone. Other significant toxicities were haematological in nature. Grade 3/4 granulocytopenia was seen in 42% of patients, while elevation of transaminase levels was seen in less than 20%. One complete response and one partial response have been seen in this trial, out of 18 patients who are evaluable at the time of writing. Another six patients have stable disease, an encouraging result in a disease that is generally resistant to treatment and in which responses tend to be infrequent.

A study of MTA in locally advanced and metastatic breast cancer is ongoing (Smith et al, 1997). Of 22 patients recruited to this study, 19 are evaluable for toxicity and 18 for response. Grade 3/4 thrombocytopenia and neutropenia were the major toxicities seen, the former in 41% of patients and the latter in 18% of patients. Other toxicities observed included grade 3/4 skin reactions in 16% of patients and grade 2/3 elevations in ALT values, seen in 84% of patients. Partial responses were seen in six patients, five of whom had previously received chemotherapy, including docetaxel, 5-FU and gemcitabine.

MTA is also being studied in the treatment of NSCLC. Two trials are ongoing, one in Canada and the other a joint South African and Australian study. The first of these, an NCIC study, has enrolled 19 patients to date, 12 of whom are evaluable for response and 15 for toxicity (Rusthoven et al, 1997). Patients included had histologically proven, stage III/IV disease and were chemonaive. As determined in the phase I trials, the starting dose for the first three patients was 600 mg m$^{-2}$, but toxicities observed at this dose led to a reduction in the dose to 500 mg m$^{-2}$. Grade 3/4 neutropenia has been seen in 32% of patients, along with elevated transaminase levels; this was shown to be transient, as seen in trials of other antifolates, such as CB 3717 (Calvert et al, 1986) and raltitrexed (Burris et al, 1994). Partial responses were seen in three patients, for an overall response rate of 33%. In the second trial, 19 patients received MTA 600 mg m$^{-2}$ once every 3 weeks (Clarke et al, 1997). Of the ten patients eligible for response assessment, three partial responses have been seen and the remaining seven patients had stable disease. The principal grade 3/4 toxicity was neutropenia, which occurred in 42% of patients. Other toxicities seen included grade 3/4 rash (17%), grade 3 nausea (8%) and grade 4 vomiting (8%). Both these trials continue to accrue patients.

Patients with metastatic colorectal cancer have also been treated with MTA in two phase II studies carried out in the USA (John et al, 1997) and Canada (Cripps et al, 1997). Prior adjuvant chemotherapy was allowed in the USA study, as long as patients had been untreated for one year before inclusion in the trial. Of the 41 patients entered into the trial, 32 had colon cancer and nine had rectal cancer. All patients were evaluable for toxicity and 17 for response. The major grade 3/4 toxicity observed was neutropenia, seen in 56% of patients, while 16% and 12% of patients experienced grade 3/4 thrombocytopenia and anaemia, respectively. Skin reactions were common, occurring in 69% of patients, but were rarely significant. A complete response was seen in one patient and partial responses in three others, while seven patients had stable disease. Of the 33 patients entered into the Canadian study, 24 were chemonaive. The recommended phase II dose of 600 mg m$^{-2}$ was given to nine patients, but this was subsequently reduced to 500 mg m$^{-2}$ in the remaining 24 patients, when several early patients experienced toxicities requiring dose reduction. One complete response and six partial responses were seen in these patients, for an overall response rate of 23% (95% CI 10–42%). Grade 3/4 neutropenia was seen in 45% of patients and grade 3/4 thrombocytopenia in 12% of patients. Grade 3 rash was seen in 40% of patients. The activity of MTA in colorectal cancer demonstrated in these studies is to be further investigated in larger phase III studies.

In conclusion, although these phase II results are preliminary, MTA appears to show promising activity in the treatment of several solid tumours, including breast, colorectal, pancreas and NSCL cancers. Further data are required before conclusions can be drawn regarding the absolute efficacy, but first indications are favourable.

© Cancer Research Campaign 1998

### THE FUTURE FOR MTA

MTA is a new antifolate with a novel pharmacological profile. Preclinical studies have shown that it has several potential modes of action, including inhibition of TS, GARFT and DHFR.

Results of single-agent phase I and II trials with MTA have shown that the most common toxicities, i.e. myelosuppression and skin reactions, were generally tolerable and manageable. The dose-limiting toxicities were usually haematological. Preliminary indications are that MTA is effective against solid tumours, including NSCLC, colorectal, breast and pancreatic cancers, and phase II trials are ongoing that will assess the efficacy of the drug against these specific tumours.

The activity of MTA in NSCLC is very encouraging, given that there has been little activity seen for other antifolates in this disease. A randomized trial comparing vinorelbine with 5-FU and leucovorin concluded that 5-FU had negligible activity against NSCLC (3%) in patients with stage IV disease (Crawford et al, 1996). Three phase II trials of edatrexate showed response rates of 32% (Shum et al, 1988), 13% (Souhami et al, 1992) and 10% (Lee et al, 1990). A subsequent phase III trial in 673 patients, which compared edatrexate, mitomycin and vinblastine (EMV) with mitomycin and vinblastine (MV), failed to show improved survival in patients treated with EMV, although the response rate in the EMV arm was higher (24% compared with 16%) (Comis et al, 1994). Myelosuppression and stomatitis were more common in patients receiving the EMV combination. In a study of trimetrexate, no major objective responses were seen in patients with stage III and IV disease (Kris et al, 1989).

It is also possible that MTA will prove to be an effective component in combination therapy and, to this end, trials are planned that will study the effects of the drug in combination with 5-FU or gemcitabine. The latter combination was suggested by research that has shown that pretreatment of HT29 colon carcinoma cells with MTA results in increased antiproliferative activity of gemcitabine (Tonkinson et al, 1996). A phase I trial is underway to investigate the combination of MTA and cisplatin in patients with solid tumours (Thoedtmann et al, 1997). Trials are also planned to investigate the effect of folates on the toxicities seen with MTA, based on the observation that animals given folate supplements were better able to tolerate treatment with MTA, with fewer side-effects (Worzalla et al, 1997). Trials are also planned for combinations with gemcitabine, irinotecan, oxaliplatin, carboplatin, doxorubicin and docetaxel, and the combination of MTA with radiotherapy will also be studied, once preclinical data have been generated.

The effect of MTA in other cancers is also under investigation. Trials are underway or planned in which MTA is given to patients with renal, bladder, cervical and oesophageal cancers, although results are not yet available from these studies. Other trials are planned in which MTA will be used to treat patients with ovarian and head and neck cancers, and the results of these, and other trials nearing completion, are awaited with interest. Initial indications suggest that MTA will find a place in the anti-cancer armamentarium.

### ACKNOWLEDGEMENTS

The authors would like to acknowledge the contribution of all the MTA investigators who have provided the results described in this manuscript, in particular the medical and nursing staff at Newcastle General Hospital: Dr M Lind, Dr N Bailey, Dr S Gokal and Dr A Hughes, F Chapman, M Proctor, D Simmons and A Oakley. Data management support was provided by L Robson and K Fishwick. We also thank Dr D Thornton, J Chick, S McCarthy and J Stickland of Eli Lilly and Co. for their assistance and Deirdre Conlon (Adelphi Communications Ltd) for assistance in the preparation of this manuscript.

### REFERENCES

Beardsley GP, Moroson BA, Taylor EC and Moran RG (1989) A new folate antimetabolite, 5,10-dideaza-5,6,7,8-tetrahydrofolate is a potent inhibitor of *de novo* purine synthesis. *J Biol Chem* **264**: 26–30

Burris H, Von Hoff D, Bowen K, Heaven R, Rinaldi D, Eckardt J, Fields S, Campbell L, Robert F, Patton S and Kennealey G (1994) A phase II trial of ZD1694, a novel thymidylate synthase inhibitor, in patients with advanced non-small cell lung cancer. *Annals Oncol* **5** (Suppl 5): 133, A244

Calvert AH, Jones TR, Dady PJ, Grzelakowska-Sztabert B, Paine RM, Taylor GA and Harrap KR (1980) Quinazoline antifolates with dual biochemical loci of action. Biochemical and biological studies directed towards overcoming methotrexate resistance. *Eur J Cancer* **16**: 713–722

Calvert AH, Alison DL, Harland SJ, Jackman AL, Jones TR, Newell DR, Siddik ZH, Wiltshaw E, McElwain TJ, Smith IE and Harrap KR (1986) A phase I evaluation of the quinazoline antifolate thymidylate synthetase inhibitor $N^{10}$-propargyl-5,8-dideazafolic acid. *J Clin Oncol* **4(8)**: 1245–1252

Clarke S, Boyer M, Milward M, Findlay M, Ackland S, Childs A, Brew S and Walcher V (1997) Phase II study of LY231514, A multitargated antifolate (MTA), in patients with advanced non-small cell lung cancer (NSCLC). *Proc Am Soc Clin Oncol* **16**: 465: A1670

Comis R, Kris MG, Lee JS, Gralla RJ, Shridhar K, Crawford J, Batist G, Wernz J, Belani C, Vokes E, Einzig A, Grunberg SE, Niedhart J, Blumenreich M, Maurer H, Evans W (1994) Multicenter, randomized trials in 673 comparing the combination of edatrexate mitomycin and vinblastine (EMV) with mitomycin and vinblastine (MV) in patients with stage IIIB and IV non-small cell lung cancer. *Lung Cancer* **11** (suppl 1): 119

Crawford J, O'Rourke M, Schiller JH, Harris Spiridonitis C, Yanovich S, Ozer H, Langleben A, Hutchins L, Koletsky A, Clamon G, Burman S, White R and Hohneker J (1996) Randomized trial of vinorelbine compared with fluorouracil plus leucovorin in patients with stage IV non-small-cell lung cancer. *J Clin Oncol* **14**(10): 2274–2784

Cripps MC, Burnell M, Jolivet J, Lofters W, Fisher B, Panasci L, Iglesias J and Eisenhauer E (1997) Phase II study of a multi-targeted antifolate (LY231514) (MTA) as first-line therapy in patients with locally advanced or metastatic colorectal cancer (MCC). *Proc Am Soc Clin Oncol* **16**: 267: A949

Farber S, Diamond LK, Mercer RD, Sylvester RF and Wolff JA (1948) Temporary remissions in acute leukemia in children produced by folic acid antagonist, 4-aminopteroyl-glutamic acid (aminopterin). *N Eng J Med* **238**: 787–793

Faries D (1994) Practical modifications of the continual reassessment method for phase I cancer trials. *J Biopharm Stat* **4**: 147–164

Gorlick R, Goker E, Trippett T, Waltham M, Banerjee D and Bertino JR (1996) Intrinsic and acquired resistance to methotrexate in acute leukemia. *Drug Ther* **335**: 1041–1048

John W, Clark J, Burris H, Picus J, Schulman L, Thornton D and Lochrer P (1997) A phase II trial of LY231514 in patients with metastatic colorectal cancer. *Proc Am Soc Clin Oncol* **16**: 292: A1038

Jolivet J, Cowan KH and Curt GA (1983) The pharmacology and clinical use of methotrexate. *N Engl J Med* **309**: 1094–1104

Kris MG, D-Acquisto RW, Gralla RJ, Burke MT, Marks LD, Fanucchi MP and Heelan RT (1989) Phase II trial of trimetrexate in patients with stage III and IV non-small-cell lung cancer. *Am J Clin Oncol* **12**(1): 24–26

Lee JS, Libshitz HI, Murphy WK, Jeffries D and Hong WK (1990) Phase II study of 10-ethyl-10-deaza-aminopterin (10-EdAM; CGP 30 694) for stage IIIB or IV non-small cell lung cancer. *Invest New Drugs* **8**(3): 299–304

McDonald AC, Vasey PA, Walling J, Lind MJ, Bailey NP, Siddiqui N, Twelves C, Cassidy J and Kaye SB (1996) Phase I and pharmacokinetic study of LY231514, the multitargeted antifolate, administered by daily × 5 q 21 schedule. *Annals Oncol* 7(Suppl 5): 126: A608

Miller KD, Loehrer PJ, Picus J, Blanke C, John W, Clark J, Shulman L, Burris H, Thornton D (1997) A phase II trial of LY231514 in patients with unresectable pancreatic cancer. *Proc Am Soc Clin Oncol* **16**: 297: A1060

O'Connor BM, Jackman AL, Crossley PH, Freemantle SE, Lunec J and Calvert AH (1992) Human lymphoblastoid cells with acquired resistance to $C^2$-desamino-$C^2$-methyl-$N^{10}$-propargyl-5,8-dideazafolic acid. *Cancer Research* **52**: 1137–1143

© Cancer Research Campaign 1998

Rinaldi DA, Burris HA, Dorr FA, Woodworth JR, Kuhn JG, Eckardt JR, Rodriguez G, Corso SW, Fields SM, Langely C, Clark G, Faries D, Lu P and Von Hoff DD (1995) Initial phase I evaluation of the novel thymidylate synthase inhibitor, LY231514, using the modified continual reassessment method for dose escalation. *J Clin Oncol* **13**: 2842–2850

Rinaldi DA, Burris HA, Dorr FA, Rodriguez G, Eckhardt SG, Fields SM, Woodworth JR, Kuhn JG, Langley C, Clark G, Lu P and Von-Hoff DD (1996) A phase I evaluation of LY231514, a novel multitargeted antifolate, administered every 21 days. *Proc Am Soc Clin Oncol* **15**: 489

Rusthoven J, Eisenhauer E, Butts C, Gregg R, Dancey J, Fisher B and Iglesias J (1997) A phase II study of the multi-targeted antifolate LY231514 in patients with advanced non-small cell lung cancer. *Proc Am Soc Clin Oncol* **16**: 480: A1728

Schultz RM, Andis SL, Bewley JR, Chen VJ, Habeck LL, Mendelsohn LG, Patel VF, Rutherford PG, Self TD, Shih C, Theobald KS, Worzalla JF, Houghton PJ (1996) Antitumor activity of the multitargeted antifolate LY231514. *Proc Am Assoc Clin Res* **37**: 380

Shih C, Chen VJ, Gossett LS, Yates SB, MacKellar WC, Habeck LL, Shackelford KA, Mendelshohn LG and Soose DJ (1997) LY231514, a pyrrolo[2,3-d]pyrimidine based antifolate that inhibits multiple folate requiring enzymes. *Canc Res* **57**: 1116–1123

Shum KY, Kris MG, Gralla RJ, Burke MT, Marks LD and Heelan RT (1988) Phase II study of 10-ethyl-10-deaza-aminopterin in patients with stage III and IV non-small-cell lung cancer. *J Clin Oncol* **6**(3): 446–450

Smith IE, Miles DW, Coleman RE, Lind MJ, McCarthy S and Chick J (1997) Phase II study of LY231514 (MTA) in patients (pts) with locally recurrent or metastatic breast cancer (LR/MBC) – an interim report. *Proc Am Soc Clin Oncol* **16**: 191: A671

Souhami RL, Rudd RM, Spiro SG, Allen R, Lamond P and Harper PG (1992) Phase II study of edatrexate in stage III and IV non-small cell lung cancer. *Cancer Chemother Pharmacol* **30**: 465–468

Taylor EC, Kuhnt D, Shih C, Rinzel SM, Grindey GB, Barredo J, Jannatipour M and Moran RG (1992) A dideazatetrahydrofolate analogue lacking a chiral center at C-6, N-(4-(2- (2-amino-3,4-dihydro-4-oxo-7H-pyrrolo(2,3-d)pyrimidin-5-yl)ethyl)benzoyl)-L-glutamic acid, is an inhibitor of thymidylate synthase. *J Med Chem* **35**: 4450–4454

Thoedtmann R, Kemmerich M, Depenbrock H, Blatter J, Ohnmacht U, Rastetter J, Hanauske AR (1997) A phase I study of MTA (multi-targeted antifolate, LY231514) plus cisplatin (cis) in patients with advanced solid tumours. *Eur J Cancer* **33** (suppl 8): S247

Thymidylate Synthase Inhibitor LY 231514. Clinical Investigation Brochure. Lilly Research Laboratories, Indianapolis, IN, Eli Lilly & Co, May 1992.

Tonkinson JL, Wagner MM, Paul DC, Gates SG, Marder P, Mendelsohn LG and Williams DC (1996) Cell cycle modulation by the multitargeted antifolate, LY231514, increases the antiproliferative activity of gemcitabine. *Proc Am Assoc Cancer Res* **37**: 370

Von Hoff DD (1988) Human tumor cloning assays: Applications in clinical oncology and antineoplastic agent development. *Cancer Met Reviews* **7**: 357–371

Ward WHJ, Kimbell R and Jackman AL (1992) Kinetic characteristics of ICI D1694: A quinazoline antifolate which inhibits thymidylate synthase. *Biochem Pharmacol* **43**: 2029–2031

Woodworth J, Rinaldi D, Burris H, Thornton D, Reddy S, Kuhn J and Von Hoff D (1997) Assessments of hemotoxicity and relationships to pharmacokinetics from a LY23514 phase I study. *Proc Am Soc Clin Oncol* **16**: 734: 210a

Worzalla JF, Self TD, Theobald KS, Schultz RM, Mendelsohn LG and Shih C (1997). Effects of folic acid on toxicity and antitumor activity of LY231514 multi-targeted antifolate (MTA). *Proc Am Assoc Cancer Res* **38**: 478

 © Cancer Research Campaign 1998